# Molecular Epidemiology of *Pseudomonas aeruginosa* in the Intensive Care Units – A Review

**DOI:** 10.2174/1874285800701010008

**Published:** 2007-09-20

**Authors:** D.S Blanc, P Francioli, G Zanetti

**Affiliations:** Hospital Preventive Medicine, University Hospital of Lausanne, Switzerland

**Keywords:** *Pseudomonas aeruginosa*, molecular epidemiology, intensive care unit

## Abstract

*Pseudomonas aeruginosa* is one of the leading nosocomial pathogens in intensive care units (ICU). This opportunist pathogen is commonly recovered from moist environments, and is also found colonizing 2.6 to 24% of hospitalized patients. We reviewed the recent literature that used highly discriminatory typing methods to precisely identify the reservoirs and modes of transmission of this microorganism in the ICU setting. In most ICUs, the endogenous flora was suspected to be the main source of infection compared to exogenous sources (other patients, the contaminated environment such as sinks or taps). However, the percentage of endogenous versus exogenous sources might vary considerably from one setting to another. Reasons for this include the compliance of health care workers to infection control measures, the contamination of the environment, and probably also the biology of the pathogen (intrinsic fitness factors). As *P. aerugi-nosa* is ubiquitous in the environment and colonizes up to 15% of hospitalized patients, eradication of the reservoir is difficult, if not impossible. Therefore, efforts should primarily focus on reinforcement of infection control measures to limit its transmission.

## INTRODUCTION

*Pseudomonas aeruginosa* is an ubiquitous environmental bacterium with minimal requirements for survival and a remarkable ability to adapt to a variety of environmental challenges. However, nearly all cases of infection occur in hosts with compromised immune defense. Nosocomial infections are known to affect most often neutropenic patients and otherwise immuno-compromized patients in intensive care units (ICUs). Given the widespread presence of *P. aeruginosa* in the environment, it is noteworthy that diseases attributable to it are quite rare in otherwise healthy individuals. Indeed, each day we contact *P. aeruginosa* by millions in our food, by thousands on the implements used to bathe us, and sometimes even in the drinking water in low numbers [1].

Although humans contact large numbers of *P. aerugi-nosa*, the species only colonize the normal human host intermittently. In contrast, it was found to be part of the intestinal flora of 2.6 to 24% of hospitalized patients [2]. However, the concentration was low (<10^2-3^ /g) and at a significant competitive disadvantage to the major endogenous normal flora, which achieves concentration of at least 10^8^/g. Nevertheless, the importance of *P. aeruginosa* as an opportunistic pathogen relies in its ability to activate useful phenotypes under environmental stress and to persist in adverse conditions such as the presence of antibiotic or antiseptic substances. The production of a slime layer provides a significant adaptation to a wide variety of adverse environmental conditions.

*P. aeruginosa* does not attack normal tissues. Intact skin and mucous membranes therefore provide an initial barrier against the attachment of the bacteria. Specific conditions must be met for the establishment of infection: the bacteria must contact the target organ in large numbers and possess certain virulence factors; the host must possess certain defects in its defense and immune system; and particular mi-croenvironmental signals must be generated to activate the bacterium (quorum sensing) [3].

Beside patients with cystic fibrosis for whom *P. aerugi-nosa* was the main death threat for several decades, risk factors for an infection with *P. aeruginosa* in the hospitalized patients include mechanical ventilation, chronic obstructive pulmonary disease, burned wounds and previous antibiotic therapy. New categories of patients susceptible to this pathogen have appeared and the number of patients in these categories is constantly increasing as new medical therapies against cancer are developed and applied and the transplantation of solid organs, skin, and bone marrow is made possible. Modern medical technology has created new specific niches for opportunist pathogens.

An infection with *P. aeruginosa* in the hospital manifests primarily as acute lung infection in ICU patients. In the European Prevalence of Infection in Intensive Care Study, up to 28% of nosocomial infections were attributed to *P. aeruginosa* [4]. In the first study on the prevalence of noso-comial infections in Swiss university hospitals, *P. aerugi-nosa* was the third agent responsible for infection (11%) following the infective agents *Staphylococcus aureus* and *Es-cherichia coli* [5].

### Source of *P. aeruginosa* Infection: Lesson from Molecular Epidemiology

As *P. aeruginosa* is ubiquitous in the environment and is also part of the endogenous flora of hospitalized patients, only studies using powerful molecular typing methods can explore the routes of colonization and/or infection. We addressed elsewhere the caution required for application of these methods [6].

The question we would like to raise here is whether *P. aeruginosa* infections in ICU patients are mainly due to endogenous or exogenous sources. In addition, when the source is exogenous, we would like also to know the respective role of patient-to-patient and environment-to-patient transmission. A better understanding of the epidemiology of *P. aeru-ginosa* in the ICU setting would allow improvement in infection control measures.

#### Evidence in Favour of Endogenous Infection

Several recent studies using molecular typing in a non-epidemic ICU setting suggested that the major reservoir of *P. aeruginosa* was the endogenous flora of the patient. These observation raised doubt on the value of barrier precautions for prevention of *P. aeruginosa* colonization or infection. In one of the first studies that investigated this topic, a German team prospectively searched for *P. aeruginosa* in patients, staff members and environment during a 4-month period in a surgical ICU [7,8]. They found a low number of patients with *P. aeruginosa* (18/153, 12%), most of which were colonized by a unique genotype, suggesting endogenous colonization (only 2 possible transmissions from patient to patient were suspected).

Berthelot *et al.* investigated the respective contribution of endogenous and exogenous transmission of *P. aeruginosa* in mechanically ventilated patients [9]. The presence of *P. aeruginosa* was prospectively examined in the respective patients and in the environment. The origin of lung colonization was endogenous in 80% of the cases (21/26).

In a Dutch ICU, Bonten *et al.* prospectively investigated the patient’s colonization and infection with *P. aeruginosa* during a period of endemicity [10]. They found that the respiratory tract colonization was of exogenous origin in only 8% of the cases.

In a similar study, Speijer *et al.* concluded that the small number of identified patient-to-patient transmissions (5 among 49 patients with *P. aeruginosa*) and the large number of genotypes found indicated that most *P. aeruginosa* strains originated from the patients themselves [11].

#### Evidence in Favour of Exogenous Infection

On the other hand, other studies have shown that transmissions from patient to patient or from environment to patient played an important role. Cross-colonization was highlighted in a study by Bergmans *et al.* [12]. They prospectively investigated the colonization and/or infection of 100 patients in two ICUs during a period of endemicity. In 16 of 23 patients with *P. aeruginosa*, a nosocomial acquisition was suspected. In another study, Thuong *et al.* conducted a prospective epidemiological investigation because of a high prevalence of *P. aeruginosa* infections in their ICU patients [13]. They found that 67% of the patients harbored an identical *P. aeruginosa* genotype, suggestive of the dissemination of an epidemic strain. Cross-colonization occurred frequently, but the role of the environment was not evaluated during this investigation. Similarly, in a 6-mounth prospective study, cross-transmission was suspected in 46% of the patients [22]. However, as no environmental or staff sampling was performed, the source of contamination could not be assessed.

The potable water was also associated with *P. aeruginosa* infection [14], especially when it was used for hydrotherapy of burn patients [15,16] or rinsing medical devices after disinfections [17]. In burn patients, Tredget *et al.* showed that hydrotherapy was a significant risk for *P. aeruginosa* infections [16].

The environment was suspected to be a non-negligible source when the aerator faucets and the sinks were highly contaminated with *P. aeruginosa* [18,19]. A recent prospective investigation over a 40-week period showed that tap water from faucets contaminated with *P. aeruginosa* played a crucial role (42% of patients infected with *P. aeruginosa* in clinical specimens) in the propagation of this pathogen not only to ICU patients, but also to patients from other adjacent wards, through patient-to-patient transmissions [20]. This investigation also showed that taps could be contaminated with the same *P. aeruginosa* genotype over an extended period of time (up to 2 years). A similar investigation was performed on intubated patients in a Spanish hospital during a 3-year prospective study [21]. The authors found that the source of strains causing ICU-acquired colonization was predominantly environmental, in most cases responsible was the tap water in the patient’s room.

It has been suggested that *P. aeruginosa* can readily contaminate staff hands, presumably by water splashed from sinks during hand washing. However, Grundman *et al.* failed to experimentally contaminate hands when sterile water was splashed directly into densely colonized sinks [23]. The authors proposed a direct transmission from the tap water to the patient (in their case by bathing neonates).

We have also investigated the hypothetical role of tap colonization with *P. aeruginosa* in ICU patients of our hospital [24,25], despite the fact that relatively few faucets of the units (12%) were colonized. A prospective molecular epidemiological investigation was performed during a non-epidemic period with a duration of one year (1998). The incidence of patients with clinical specimens positive for *P. aeruginosa* was 55.7 infected patients (cases)/1000 admitted patients (admissions). We found that 42% of the cases (23.6 cases/1000 admissions) had isolates identical to those found in the taps. Although the initial contamination of the taps could originate from retrograde colonization, most of the *P. aeruginosa* genotypes (82%) were recovered from the taps before they were isolated from patients. This finding strongly suggests that the water system was the primary reservoir. Among patients with isolates that were not found in the taps, 30% (16.5 cases/1000 admissions) had isolates identical to those of at least one other patient hospitalized during the same period in the same unit, suggesting a possible patient-to-patient transmission. In the remaining 28% of the cases (15.6 cases/1000 admissions), the isolates showed a unique typing pattern, indicating that the source was probably endogenous. Control measures were taken in order to reduce i) the presence of *P. aeruginosa* in taps, ii) the use of tap water for certain patient care procedures (replacement of tap water by *P. aeruginosa*-free bottled water for drinking and mouth wash of patients), and iii) the transmission from patient to patient. In 2000, the same molecular epidemiologi-cal investigation was done [26]. Despite the fact that the same proportion of taps contaminated with *P. aeruginosa* was observed, the incidence of patients with *P. aeruginosa* from exogenous source (identical to isolates from the taps or to another patient) decreased by a factor of 3 (13.5 cases/1000 admissions in 2000 versus 40.1 cases/1000 admissions in 1998), whereas the incidence of patients with isolates showing a unique pattern remained the same (13.1 cases/1000 admissions in 2000 versus 15.6 in 1998). As the decrease of cases was similarly observed in both patients with isolates from the taps and patients with isolates identical to other patients, and as the eradication of *P. aeruginosa* in the taps did fail, the main effect of the infection control measures relies probably on the transmission of the pathogen. The principal measures that were responsible for this effect might be the introduction of an alcoholic solution for hand disinfections and the reinforcement of standard precautions. Similar results were obtained by Trautmann *et al*. who showed that during a one-year period of time, 50% of patients infected or colonized with *P. aeruginosa* shared a similar genotype that the one found in tap water [27].

Although most *P. aeruginosa* infections are endemic, outbreaks have also been described and contribute to the extent of infections of exogenous origin. Recent reports of *P. aeruginosa* outbreaks were due to multidrug-resistant genotypes, which complicate the treatment of infections. Bert *et al.* reported an outbreak of multi-resistant *P. aeruginosa* involving 36 patients in a neurosurgery ICU [28]. The strain was cultured from enteral nutrition solutions as well as from tap water and sinks. Control of the outbreak was possible after reinforcement of isolation procedures for infected patients, modification in the mode of enteral nutrition and replacement of all sinks in the unit. Concern about multidrug-resistant strains was also highlighted in the report by Hsueh *et al.* who traced the spread of a single strain of *P. aerugi-nosa* over a period of several years [29]. Berthelot *et al.* also reported the occurrence of one multi-resistant genotype in patients and in sinks during their prospective investigation of *P. aeruginosa* in ICU patients (9). The situation resolved when chlorine disinfection of sinks was reinforced.

## CONCLUSIONS

**Fig. (1):**
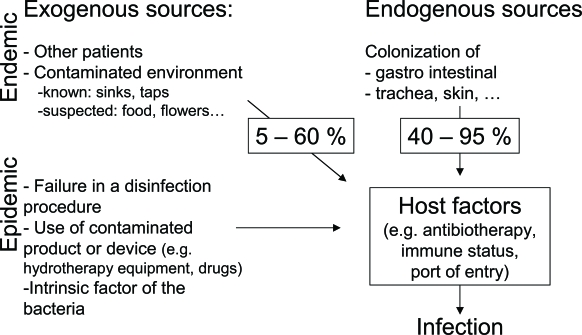
Schematic representation of the factors contributing to the epidemiology of *Pseudomonas aeruginosa* in the Intensive Care Units. Other patients or contaminated environment are potential exogenous sources of infections. Failure in a disinfection procedure, the use of contaminated product or device are often cited as the source of epidemics. Intrinsic factors of the bacteria (e.g. multidrug-resistance) might also play a role in epidemics. On the other hand, colonization of the gastro-intestinal track, the trachea or the skin are the major endogenous sources of infections. Infections in the patients will occurred only if an opportunity is given to the pathogen (e.g. antibiotic therapy, immuno-supression, port of entry).

Review of all these molecular investigations on the epidemiology of *P. aeruginosa* in the ICU setting shows that the contribution of endogenous versus exogenous reservoirs to the colonization and infection of patients varies according to the compliance of health care workers to infection control measures, to the contamination of the environment, and probably also to the biology of the pathogen (intrinsic factors) (Fig. 1). Nevertheless, as the environment was found to play an important role in several investigations, the question is, should we eradicate this reservoir, and how? Taps contaminated with *P. aeruginosa* were shown to serve as a continuous source for transmission [20,27], but, it appears almost impossible to eradicate this contamination. One possible option might be the use of microfiltres at each tap (end line filtration) [30,31]. Regular disinfection of contaminated sinks was shown to reduce the number of nosocomial infections due to *P. aeruginosa* [32] and was able to eradicate multi-resistant strains [9]. On the other hand, in our investigation [25], control measures against the transmission of the pathogen, e.g. alcoholic solution for hand disinfection, were probably responsible for the decrease of *P. aeruginosa* infections in ICU patients.

As *P. aeruginosa* is ubiquitous in the environment and colonizes up to 15% of hospitalized patients, eradication of the reservoir is difficult, if not impossible. Therefore, efforts should primarily focus on the reinforcement of infection control measures to limit the transmission of *P. aeruginosa*. However, when a multi-resistant strain is repetitively recovered from patients and from the environment, efforts should be undertaken to achieve eradication of this strain from the environment.
